# Supplementary figures and images for: A Functional Carbohydrate Degrading Enzyme Potentially Acquired by Horizontal Gene Transfer in the Genome of the Soil Invertebrate Folsomia candida
Source: Genes (Basel). 2022 Aug 7;13(8):1402. doi: 10.3390/genes13081402 (PMC9460274; doi:10.3390/genes13081402)

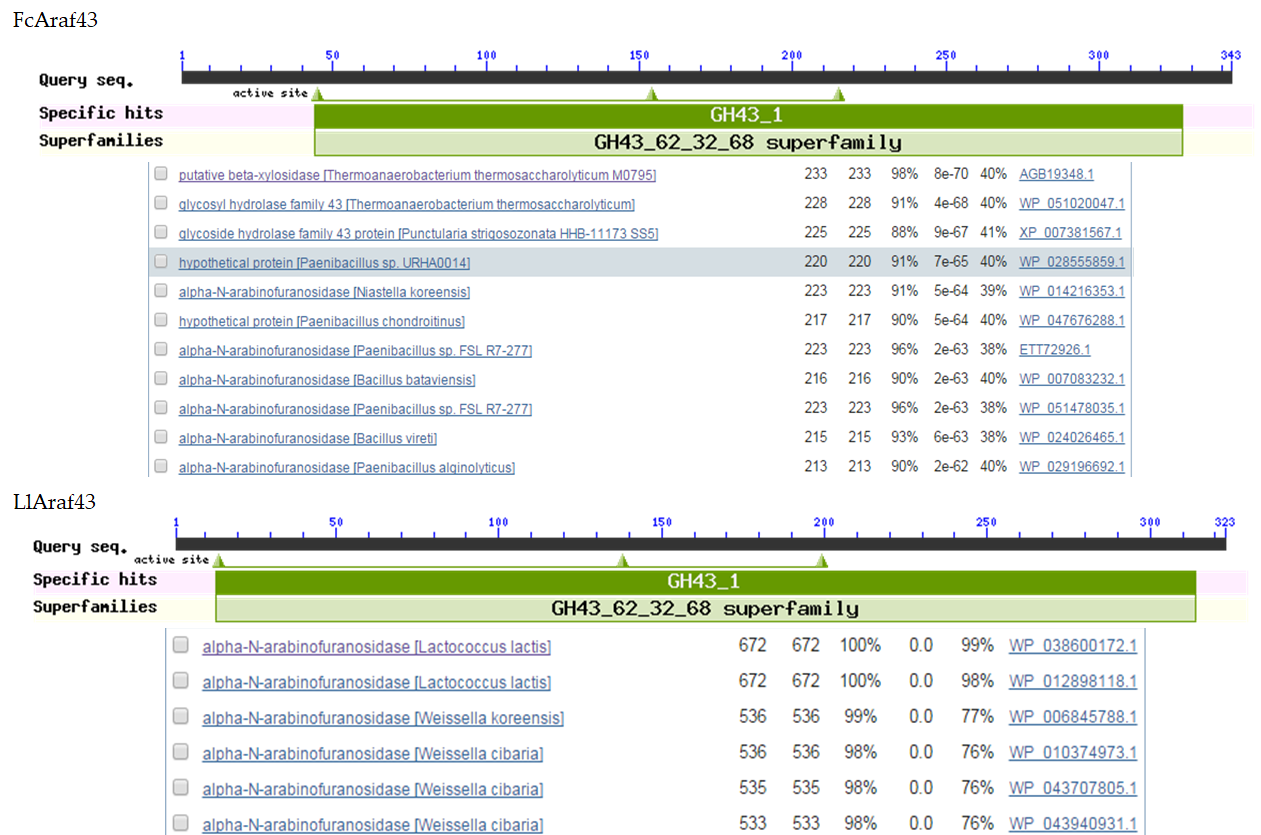

Supplement: Supplementary file 1 [file genes-13-01402-s001.zip › supplementary Figure S1.png]

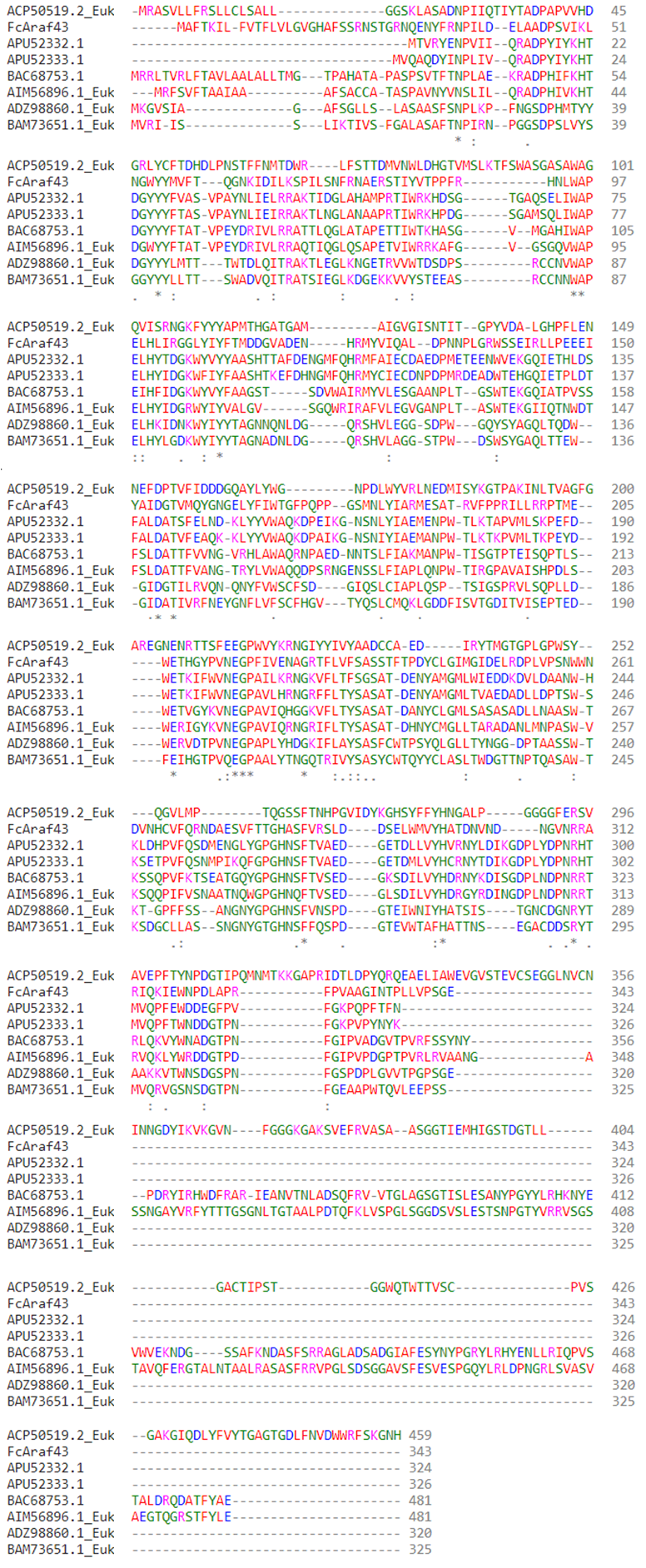

Supplement: Supplementary file 1 [file genes-13-01402-s001.zip › supplementary Figure S2.png]
